# Realist assessment of fidelity during the implementation of the PARTNERS collaborative care intervention for people with diagnoses of severe mental illness within a cluster randomised controlled trial

**DOI:** 10.1371/journal.pmen.0000130

**Published:** 2024-11-15

**Authors:** Charley Hobson-Merrett, Julia Frost, Ruth Gwernan-Jones, Vanessa Pinfold, Michael Clark, Shamiaa El Naggar, Linda Gask, Bliss Gibbons, John Gibson, Siobhan T. Reilly, Debra Richards, Angela Saunders, Debs Smith, Richard Byng

**Affiliations:** 1 Community and Primary Care Research Group, University of Plymouth, Plymouth, United Kingdom; 2 Health and Community Sciences, University of Exeter, Exeter, United Kingdom; 3 Independent; 4 The McPin Foundation, London, United Kingdom; 5 Care Policy and Evaluation Centre, The London School of Economics and Political Science, London, United Kingdom; 6 Division of Population Health Sciences and Primary Care, University of Manchester, Manchester, United Kingdom; 7 Coventry and Warwickshire Partnership NHS Trust, Coventry, United Kingdom; 8 Centre for Applied Dementia Studies, University of Bradford, Bradford, United Kingdom; IRCCS Centro San Giovanni di Dio Fatebenefratelli, ITALY

## Abstract

Many with severe mental illnesses are underserved by disjointed service provision. PARTNERS aims to address this via collaborative care with recovery-based coaching. PARTNERS was evaluated in a randomised controlled trial. Understanding how intervention delivery compared to the model, why this was, and under what circumstances, aids interpretation of trial results and optimisation of future implementation. This paper reports the results of a Realist assessment of fidelity, exploring delivery compared to model and refining programme theory. Practitioners, service users, supervisors, primary care representatives, and researchers (n = 39) were interviewed. Additional data included session recordings, follow up interviews, practitioner reflective logs, supervision logs, contact data, service user surveys, and meeting minutes. A framework analysis with evaluative coding was used to assess the extent to which delivery matched the Realist initial programme theory, and how, why and under what circumstances this was the case. Retroductive analysis was used to refine the programme theory. Delivery was good, but varied by practitioner and over time. Delivery improved over time, as practitioner understanding of the intervention increased. Refinements to the programme theory include training leading to practitioners forming collaborative relationships with service users most of the time, but unidentified contextual factors causing variation in consistency. Whether training led to practitioners liaising across different bodies was dependant on the contextual factors of existing relationship skills and previous connections. System-level difficulties in providing consistent supervision made it difficult to assess the impact of this mechanism on delivery. Variation in delivering means caution should be applied when interpreting trial results. Implementation of practitioner-level change without implementing system-level change limits the ability to fully implement the model and to draw conclusions as to effectiveness. Current changes to NHS community mental health care may make this more achievable. Further research is needed to understand the role of supervision and optimal training.

**Trial registration:** This is the realist process evaluation of the cluster randomised controlled trial ISRCTN95702682.

**REC approval:** West Midlands–Edgbaston Research Committee 29/06/2017, ref: 14/WM/0052 (trial registration number ISRCTN95702682).

## 1. Background

There is increasing pressure in England to join-up care for people with severe mental illness (SMI) [1, 2]. However, policies offer little detail as to what care should be delivered or how plausible it is to deliver new models of working. Collaborative care (CC) is a potential organisational and clinical approach to tackling these problems, and has a good evidence base for depression [3]. Personal recovery approaches [4, 5] are also advocated, with evidence that these can improve psychosocial outcomes via increased hope, agency, and purpose [6, 7]. Coaching has been identified as one approach to supporting recovery in those with significant mental health needs [8, 9] and has been used as part of CC for depression. The PARTNERS intervention is unique in using a recovery-oriented coaching approach as the therapeutic modality of CC for individuals with SMI diagnoses.

### 1.1 The PARTNERS intervention

PARTNERS was developed and tested in the PARTNERS2 research programme. PARTNERS is a complex intervention developed and refined using Realist evaluation approaches [10, 11]. In PARTNERS a practitioner (the ‘care partner’ (CP)) with experience of caring for individuals with mental health problems works collaboratively with the service user (SU), liaising with primary care, secondary care, tertiary services, and friends and family when appropriate. They create a shared understanding of what is important to the SU and utilise coaching to support the SU to make personalised positive changes to their lives. To enable cross-service liaison, the CPs are located in GP practices, and are employed and supervised by secondary care. The PARTNERS intervention is guided by a manual and training, and includes Gunn et al.’s ]3] four elements of CC: Multi-professional approach including primary care; Structured management plan in the form of a handbook; Scheduled follow-ups; Enhanced inter-professional communication). The PARTNERS intervention is expected to interact with and improve hope, agency, identity, and quality of life for people with SMI diagnoses. The Realist initial programme theory underpinning this has two levels: the CP and SU levels, with outcomes from the former becoming the resources in the mechanisms of change in the SUs level programme theory. Briefly, in the CP level of the initial programme theory:

- Initial and ongoing training, a handbook (available on request by email), and tape assisted recall are provided to the CP and their secondary care supervisor. These resources, alongside secondary care engagement and peer supervision create the following changes of reasoning in the care partner:
  ▪ Care partner understands and engages with the intervention;
  ▪ Care partner understands how and has the skills to deliver the intervention;
  ▪ Care partner feels supported and confident to deliver the intervention and manage risk.
- The above mechanism leads to the outcome of the CP providing the following to the SU:
  ▪ Collaborative style of interaction;
  ▪ Proactive engagement;
  ▪ A shared understanding between the CP and the SU of what is important to the SU;
  ▪ Psychosocial intervention in the form of coaching and goal setting;
  ▪ Liaison with primary care, secondary care and the third sector;
  ▪ Liaison with friends and family.
- The extent to which the above mechanism works is dependent on the contextual factors of CP existing skills, knowledge, and experience, including communication skills and previous work within a mental health setting. Mechanisms are more likely to create outcomes if the CP has greater existing skills, knowledge and experience.

**Fig 1:**
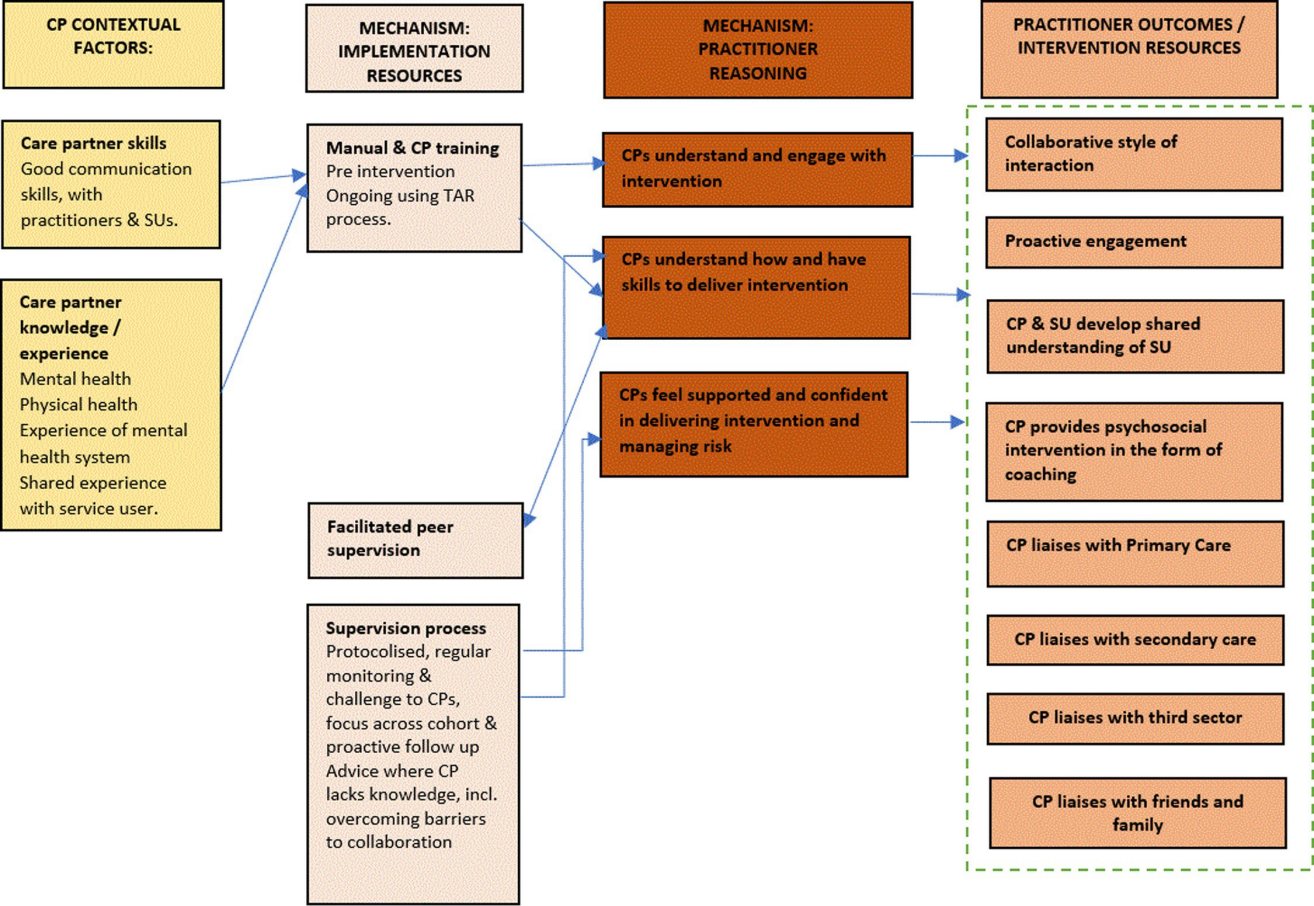
Initial CP programme theory. Note: The practitioner outcomes in the CP level theory become the SU resources in the SU level theory.

The CP level programme theory is also described in Fig 1.

**Fig 2:**
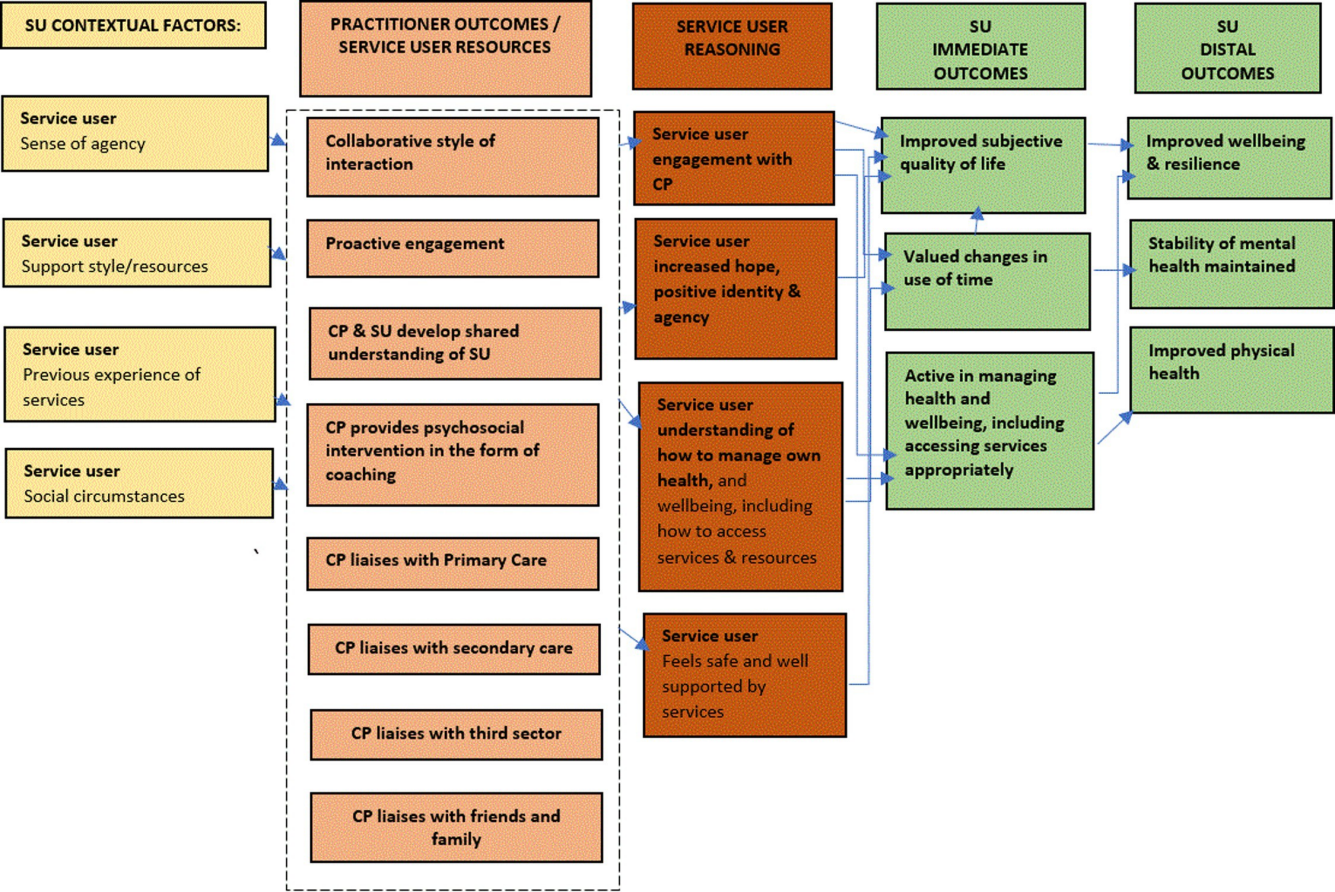
Initial SU programme theory. Note: The practitioner outcomes in the CP level theory become the SU resources in the SU level theory.

The SU level programme theory describes how, why, and under what circumstances the outcomes in the CP level programme theory become resources that create positive changes for SUs, including improved quality of life (see Fig 2).

The clinical and cost-effectiveness of the PARTNERS intervention was tested via practice-based cluster randomised controlled trial for people with lower needs and either discharged to primary care or still under specialist services [12, 13]. CPs delivered the PARTNERS intervention between August 2018 and February 2021, with SUs receiving up to 12 months of support. The trial returned a neutral result, with no difference between arms.

Exploring the fidelity of the PARTNERS intervention as delivered in the trial is fundamental to understanding to what extent the trial results reflect the effectiveness of the planned intervention, and for planning any further implementation of PARTNERS. The exploration of fidelity in complex interventions such as PARTNERS, which require practitioner decision making and individualised care, is not well suited to purely quantitative approaches [14–17]. For example, PARTNERS requires practitioners to make decisions on how to provide individualised care for each person, based on a shared understanding of what is important to the person. Whether a practitioner has delivered this with good fidelity would be difficult to explore using variables, checklists and quantitative analysis, but it better suited to qualitative analysis.

Indeed, as part of The RAMESES II Project, Greenhalgh et al. [18] state that in complex interventions fidelity needs to be articulated in terms of the underpinning programme theory, in order to understand variations in fidelity due to contextual factors such as individual practitioner experience and qualification, supervision, and the local healthcare system. Therefore, this paper explores the extent to which delivery in the PARTNERS trial matched that expected by the Realist initial programme theory, including contextual factors leading to any variations in delivery of CP level outcomes; creating an understanding of how, why, for whom, and under what circumstances delivery more closely matched the existing programme theory. This Realist approach which explores the role of context on fidelity is also useful in creating an understanding of how fidelity might be improved in further implementation of the intervention. This also offers an opportunity to refine the CP level of the Realist programme theory. A refinement of the SU level Realist programme theory following the trial, including how, why and under what circumstances SU level outcomes are created is reported elsewhere.

### 1.2 Aim

This Realist assessment of fidelity took place within the context of the randomised controlled trial of the PARTNERS intervention. It aims to explore the extent to which CPs delivered the model as expected, including how, why, for whom, and under what circumstances delivery varied, and a refinement of the CP level programme theory. This Realist evaluation meets the RAMESES II realist evaluation reporting standards (see S1 Checklist).

## 2. Materials and methods

### 2.1 Setting

This study took place within the intervention arm of the PARTNERS2 cluster randomised controlled trial [12]. Twenty practices (116 SUs) were randomised to the intervention. SU inclusion criteria for the trial were: a diagnosis of bipolar, schizophrenia, or other psychosis; over 18; seen either only in primary care or if seen in secondary care considered low risk (operationalised as clusters 11 or 12 [19], or equivalent). Participants seen only in primary care needed to have some SMI mental health need recorded in their medical notes in the previous 12 months. Potential SU participants who were severely physically or mentally unwell, with severe drug or alcohol use, currently in an acute setting, or with a learning disability were excluded. Ethical approval for the trial and process evaluation (including this assessment of fidelity) was granted by the West Midlands–Edgbaston Research Committee 29/06/2017, ref: 14/WM/0052 (trial registration number ISRCTN95702682).

### 2.2 Participants

During the two-year period of intervention delivery nine practitioners filled five CP posts across four secondary care sites; all were invited to take part in this study, providing both qualitative and quantitative data. As a part of the trial follow-up data, all SUs in the intervention arm were invited to complete a survey regarding the intervention they received. Subsamples of the SUs in the trial intervention arm and their friends/family (informal carers), primary and secondary care representatives, CP supervisors, and the research team were invited to take part in qualitative interviews.

#### 2.2.1 Sampling of participants

Purposive sampling of SUs aimed to represent all CPs, and explore the heterogeneity of SU demographic characteristics and intervention time points; this was designed to enhance capture of contextual factors affecting causation. Theoretical sampling of SUs was undertaken in later data collection points to further explore aspects of programme theory identified as under-theorised during interim analysis. Researchers were invited to be interviewed based on their involvement in providing training, supervision, and research processes that formed part of the mechanisms in the programme theory. A convenience sample of primary and secondary care representatives was invited. SUs who mentioned informal carers were asked if they felt comfortable inviting that person to take part.

#### 2.2.2 Recruitment

All fidelity study participants were provided with participant information sheets and given the opportunity to ask questions before giving informed written consent. During COVID-19 restrictions, and with ethical approval, verbal consent was audio recorded and transcribed. The subsample of trial participants was identified by the research team, in consultation with the CPs. CPs initially approached SUs about taking part before the research team contacted the SU with more information and to ask for informed consent. During COVID-19 restrictions SUs gave informed consent for session recordings to CPs, instead of researchers. SUs who had withdrawn from the intervention but consented to remain a part of the trial were identified from trial data and approached by CHM. SU participants were offered a £10 shopping voucher per interview and session recording. Recruitment for the main trial ran began in February 2018, recruitment to the fidelity evaluation ran from March 2019 until December 2020.

### 2.3 Data collection

Interviews (n = 39) were undertaken by CHM, RGJ, JF, BG, JG, DR, LW and RD, overseen by JF. CHM and RD facilitated the collection of records completed by CPs.

Semi-structured interviews addressed participants’ experiences in delivering, receiving, and/or supporting the PARTNERS service. Topic guides were developed by the research team, with input from the PARTNERS2 Lived Experience Panels (LEAPs) [20]. These topic guides aimed to capture the constituent elements of the initial programme theory, allowing for each element to be assessed for delivery against model, and to explore how, why, and under what circumstances delivery might have varied.

Intervention sessions (n = 10) were recorded as both an independent data source and for tape-assisted recall interviews (TARs) [21, 22]. In TARs extracts that aligned closely with the model, diverged strongly from the model, or prompted questions from the researchers were selected to be played back to CPs and SUs in semi-structured interviews. Participants were interviewed about their intentions and reactions during each extract. These provided an additional source of data and an extra dimension of triangulation to improve rigour and reduce researcher assumptions [21].

CPs were asked to keep monthly reflective practice logs, supervision sessions logs, and to record brief details about the frequency, duration, and content of each session with all SUs on their PARTNERS case load. Templates for these records were provided in the CP manual.

As a part of the main trial, all SUs in the intervention arm were asked to complete a survey during trial follow-up. The survey listed key elements from the CP level initial programme theory outcomes, and asked SUs to state if they had received these elements. This data source provided a more superficial overview across the whole intervention cohort. Although it does not form the main focus of this paper, it was useful in exploring the extent to which the findings in the qualitative sub-samples were likely to mirror the experiences of the SU cohort as a whole.

### 2.4 Analysis

Interview and session data were transcribed by a General Data Protection Regulation compliant transcriber and anonymised. To maintain contextual integrity place names and bodies were given identifiers rather than redacted. Pseudonyms were allocated to participants for the purpose of reporting results [23].

Analysis was undertaken by CHM, JF and RGJ. Data were analysed both to create a Realist assessment of fidelity and to refine the Realist programme theory at CP level; exploring the extent to which CP level outcomes occurred, and the extent to which these outcomes were caused by the mechanisms and contextual factors predicted in the CP level initial programme theory. Table 1 describes the elements of the CP initial programme theory that were evaluated.

**Table 1:** Description of the CP initial programme theory that were explored for fidelity.

Practitioner outcomes/intervention resources explored for fidelity	Example indicators of fidelity (data were analysed for evidence of these indicators that practitioner outcomes were delivered)
Collaborative style of interaction	*CP and SU working together. *The CP is not dictating to the SU, or telling the SU what to do.
Proactive Engagement	*Where/if the SU misses sessions the CP works to understand why this is and make it easier to attend. *A move away from a ‘discharge’ model, where if you do not attend a certain amount of sessions you are removed from the service.
CP & SU develop a shared understanding of SU	*The CP has worked to knows what is important to the SU and why it is important * The CP continues to work to update this understanding * The CP and SU understanding of what is important to the SU broadly agree
CP provides psychosocial intervention in the form of coaching	* The CP works from a Recovery orientated position * The CP works with the SU to identify goals that are important to the SU * The CP works with the SU to achieve goals that are important to the SU, focusing on their strengths and potential
CP liaises with primary care and secondary care	*The CP shares the shared understanding of the SU with primary and secondary care (with SU permission) * The CP contributes to primary and secondary care records * Where the SU consents/asks the CP to: The CP works with primary and secondary care on physical health concerns, risk management, medication reviews, physical health checks *This is appropriate to the shared understanding and collaborative relationship: providing more/less support to SU in accessing these services dependant on the SU requirements, helping without ‘doing for’ or working ‘mechanistically’
CP liaises with third sector	*Where shared understanding and/or goal setting suggest helpful: the CP liaises with third sector. *This is appropriate to the shared understanding and collaborative relationship: providing more/less support to SU in accessing these services dependant on the SU requirements, helping without ‘doing for’ or working ‘mechanistically’
CP liaises with SU friends and family	*CP meets/works with/keeps informed SU friends and family (with SU permission) *This is appropriate to the shared understanding and collaborative relationship: providing more/less support to SU in accessing these services dependant on the SU requirements, helping without ‘doing for’ or working ‘mechanistically’
**Practitioner reasoning**	**Example indicators of fidelity (data were analysed for evidence of these indicators that changes in practitioner reasoning had taken place)**
CP understands and engages with the intervention	*CP describes the purpose and delivery of the intervention correctly *CP language and actions demonstrates they are aligned with the ethos of the intervention *CP tries to deliver the intervention as envisaged
CP understands how to and has the skills to deliver the intervention	*CP delivers the intervention as envisaged *CP delivers the intervention envisaged with a range of SUs *CP delivers the intervention in a flexible manner appropriate to the individual SU
CP feels supported and confident in delivering the intervention and managing risk	*CP is comfortable and confident in delivering the intervention, utilising supervisor and peer support where appropriate *CP manages risks as they arise, working with other practitioners and supervisor where appropriate
**Implementation support (resources)**	**Example indicators of fidelity (data were analysed for evidence of these indicators that the resources were provided)**
Manual & CP training	*CP attends CP training *CP receives and utilises manual *CP engages in the ongoing meta-supervision using Tape Assisted Recall to reflect on and improve their practice *CP engages in reflective practice to continually improve their own practice, using templates within the manual *CP’s supervisor receives CP training and manual
Facilitated peer supervision	*CP attends regular peer supervision with other CPs *CP works with peers to improve their own and others’ delivery of the model
Supervision process	*CP meets regularly with a supervisor to reflect on their own practice *CP engages in reflective practice with their supervisor, using templates within the manual *Supervisor supports CP to ensure they are delivering the model as expected, by helping them to overcome barriers and/or fill in knowledge gaps
**CP contextual factors**	**Example indicators of fidelity (data were analysed for evidence of these indicators that these CP contextual factors affected the extent to which the intervention resources led to the practitioner outcomes)**
CP skills	*CP existing communication skills with SUs, including: ability to listen and understand the needs, wants and interests of different SUs; ability to address power imbalances between themselves and the SU. *CP existing communications skills with PC practitioners. *CP existing communication skills with SC practitioners. *CP understanding of the pressures and working practices within primary care, enabling a better understanding of how best to communicate with primary care *CP understanding of the pressures and working practices within secondary care, enabling a better understanding of how best to communicate with secondary care
CP knowledge and experience	*CP knowledge of severe mental illness *CP experience of delivering coaching *CP knowledge of the recovery model *CP experience of working collaboratively with SUs *CP qualifications *CP NHS banding *CP previous experience of working with primary care *CP previous experience of working with secondary care *CP knowledge of local third sector providers

All above data were used to create case studies for individual CPs. Case studies were used to assess delivery compared to the PARTNERS intervention, and to understand how, why and under what circumstances delivery matched/deviated from the programme theory. To do this coding frameworks were created using the Realist context-mechanism-outcome configurations from the initial programme theory. Evaluative coding [24, 25] was used to indicate the extent to which adherence to the mechanisms and CP level outcomes in the model was supported by the data; extracts were marked with a + or–according to whether they provided evidence that delivery did or did not match the model. To undertake the evaluative coding data sources were analysed qualitatively looking at descriptions, actions, and language that were indicative of working in a way aligned with the shared ethos and broad approaches of the model. Data were also analysed inductively and deductively for indications of causes of variation in delivery. Narrative summaries were then produced in relation to each mechanism and outcome, and the case study overall. CP and SU contextual data were captured on a separate framework and analysed alongside narrative summaries to explore contextual reasons for deviations from delivery and/or variations in delivery, including investigating any researcher Realist ‘hunches’. Visual illustrations of delivery compared to model for each CP were produced [26]. Cross-case study analysis of the narrative summaries identified demi-regularities that enabled a Realist assessment of overall delivery compared to model. This also enabled a refinement of the CP level programme theory, by exploring demi-regularities in relation to how, why, and under what circumstances delivery did or did not match the model. Stakeholder meetings with the wider research team (including researchers with lived experience of mental health problems), co-applicants, and LEAP members helped to understand SU, clinical, and policy implications. Throughout this analysis it was assumed that mechanisms function on a continuum [27], rather than an on/off state, therefore analysis of delivery must be approached in the same way (e.g., a practitioner can be provided with supervision that is neither perfect nor non-existent). Additionally, it was assumed that mechanisms consist of constituent resources and changes in reasoning, and that contextual factors can act directly on both resources and reasoning [27]; this is consistent with the approach taken in the initial programme theory.

### 2.5 COVID-19

Eighteen months into the 29-month delivery of this intervention England entered a national lockdown which necessitated a change in intervention delivery that continued until the end of the study: CPs worked remotely; Face-to-face meetings with SUs were replaced with phone and/or video calls. The impact of COVID-19 on delivery is explored in a separate paper [28].

### 2.6 Patient and public involvement

Patient and public involvement was integral across the PARTNERS2 research programme, via Lived Experience Advisory Panels (LEAPs), and SU researchers [20]. Within the process evaluation LEAP members contributed to the design of the interview topic guides. Researchers who undertook data collection included SU researchers. LEAP members and SU researchers contributed to the interpretation of analysis, and to the writing of this paper.

## 3. Results

Table 2 shows participants recruited. The data from these participants was also used to test and refine the SU level initial programme theory; this is the subject of a separate paper. Table 3 shows data collected per CP. Pseudonyms have been used throughout the results section when identifying both SUs and CPs.

**Table 2:** Study participants.

Participant Type	Number of participants
CP	8 (of 9 invited)
SUs	13
CP supervisors	4
Representatives of primary and secondary care	9
Research team members	4
Family/friend of SU	1

**Table 3:** Process evaluation data collected for each of the care partners delivering PARTNERS intervention.

Care partner (months in post)	Elijah (14)	Jack (19)	Nora (21)	Sarah (12)	Tina (2)	Becky (16)	Grace (24)	Hannah (5)
Reflective practice logs	0	3	14	5	0	1	12	4
Contact sheets	All	All	All	All	None	Some	All	All
Supervision logs	0	2	10	3	7	8	8	5
Session recordings	0	2	4	0	0	0	3	1
CP follow up interviews	0	2	4	0	0	0	3	1
SU follow up interviews	0	2	4	0	0	0	3	1
CP interviews	1	1	1	1	0	1	2	0
SU interviews	0	3	2	0	1	1	3	0
Supervision interviews	0	1	1	0	0	1	1	1
GP interviews	0	2	1	0	0	0	1	0
Secondary care interviews	Five interviews with practitioners, managers and researchers							
Researcher interviews	Four researcher interviews: trainer clinician, PI clinician, Research fellow, programme manager							

CP duration in post varied considerably (range 2–24 months). Consequently, the availability, timeliness, quality, and completeness of data varied by CP. Therefore, this paper draws more heavily on the results of two contrasting CPs (pseudonyms: Grace and Nora), and the sampled SUs they worked with; this is used to illustrate the breadth of the analysis and scope of practitioner work. These CPs were in post longest, and they represent the range of previous experience within the CP cohort. Both practitioners have several years’ experience supporting those with mental health difficulties, but the difference in the nature of this experience is representative of the differences within the CP cohort: Grace has experience as a senior mental health practitioner, and Nora is an experienced support worker. These two CP case studies represent the variation in fidelity found in the overall analysis, including how, why and under what circumstances fidelity varied. Other CP case studies have also been referenced to broaden observations from the Nora and Grace analyses, particularly where the findings in relation to these two CPs was not indicative of the CP cohort as a whole.

**Fig 3:**
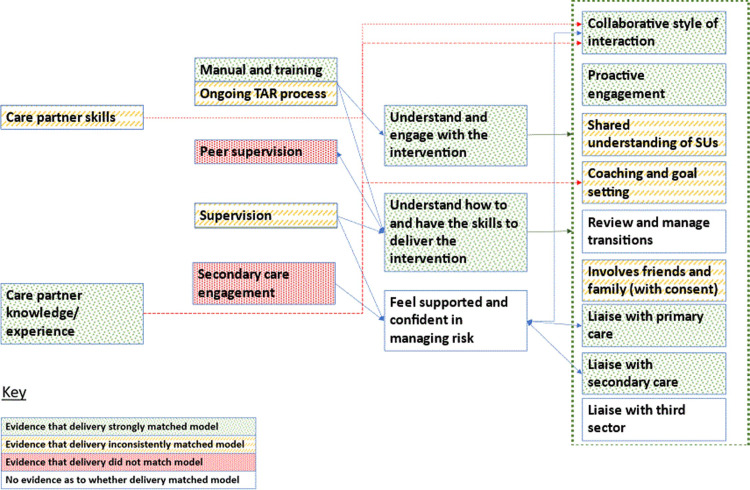
The extent to which delivery matched model for CP Grace.

**Fig 4:**
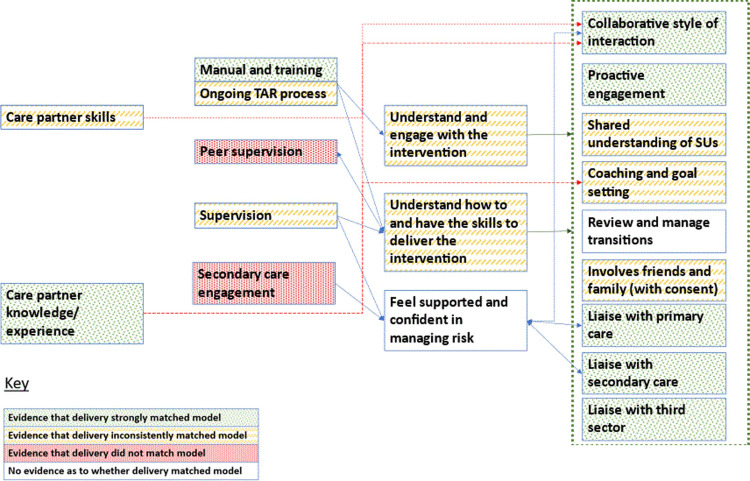
The extent to which delivery matched model for CP Nora.

Analysis highlighted variable delivery. The extent to which Nora and Grace’s delivery matched the programme theory is summarised visually in Figs 3 and 4, showing how outcomes, mechanisms and contextual factors compared to optimal delivery. For example, whilst Nora and Grace both received optimal initial training, Grace was able to engage more in ongoing tape assisted recall training; although Grace was able to consistently work collaboratively with SUs, Nora was only able to do this with some SUs. It should be noted that in all CP case studies there were difficulties in recruiting, training, and retaining supervisors. Whilst this reflects national NHS staffing pressures, it limits the analysis of the supervision and secondary care engagement resources.

The rest of the results explore in more detail how, why and under what circumstances fidelity to model varied, focusing on the causation of three CP level outcomes in the initial programme theory (creating collaborative relationships with service users, coaching and goal setting, liaising with other services) and one refinement to mechanisms that cuts across the causation of all outcomes (time to develop practice). Refinements to the CP level programme theory are also outlined in Table 4.

**Table 4:** Refinements to care partner level programme theory.

Original programme theory extract (c = context, res = resource, rea = reasoning, o = outcome)	Refinements to programme theory (c = context, res = resource, rea = reasoning, o = outcome)
If practitioners with good communication skills and experience working in mental health (c) are provided with the intervention resources (res) they will engage with, understand how to, and have the skills to (rea) work collaboratively with SUs (o).	An **unknown** CP contextual factor makes it difficult for some practitioners to individualise the intervention to work collaboratively with all SUs. This includes some CPs finding it harder to work collaboratively with SUs of higher socio-economic status. This causes variation in fidelity across CPs. *If practitioners [**unknown contextual factor*, *possibly relating to practitioner and/or SU socio-economic status] (c)* *are provided with the intervention resources (res) they will engage with*, *understand how to*, *and have the skills (rea) to work collaboratively with SUs (o)*.
If practitioners who have existing communication skills and experience working in mental health services (c) are provided with the intervention resources (res) they will engage with, understand how to, and have the skills to (rea) work with the SU using a goal setting and coaching model (o).	There is an additional context-mechanism-outcome configuration in the form of a feedback loop between the collaborative relationship and shared understanding outcomes and the coaching model outcome: *The collaborative relationship and shared understanding outcomes (o) become resources (res) that help the CP understand how to tailor (rea) delivery of the coaching outcome (o)*
If practitioners who have existing communication skills and experience working in mental health services (c) are provided with the intervention resources (res) they will engage with, understand how to, and have the skills to (rea) liaise with other services as appropriate according to the shared understand and collaborative relationship between the SU and CP (o).	It is **unclear** to what extent and under what circumstances the purpose of liaisons undertake sit within the ethos of PARTNERS: are they appropriate according to the shared understanding and collaborative relationship? **Refinement to contextual factors that facilitate this mechanism, creating variation in in fidelity across CPs:** *If practitioners with existing skills* *of being able to liaise across NHS hierarchies* *(C)…* *If practitioners with existing* *relationships across different NHS bodies* *(C)…* *Exact job previous job role/training/hierarchy is not an indicator of existing skills of being able to liaise across hierarchies or existing relationships across different NHS bodies*.
If practitioners who have existing communication skills and experience working in mental health services (c) are provided with the intervention resources (res) they will engage with, understand how to, and have the skills to (rea) deliver the PARTNERS intervention (o).	**Refinement to resource provision that creates variation in fidelity across time and across CPs:** *If* *system turnover and recruitment allows* *(c)* *practitioners to have sufficient time to practice and fully grasp the model* *(res) then they will fully understand the whole model (rea) to the extent that they can deliver its many constituent parts (o)*. *Practitioner existing skills and experience (c) facilitates* *understanding (rea) of the parts of the intervention* *(o)* *that most closely align with previous skills and experience*. *Practitioner previous experience working in mental health (c)* *makes it harder* *to understand how and have the skills (rea) to work collaboratively with SUs and develop a shared understanding with SUs (o)* *Practitioner* *previous experience of reflective practice* *(c) facilitates understanding how to and having the skills (rea) to work collaboratively with SUs and develop a shared understanding with SUs (o)*

### 3.1 Creating collaborative relationships with SUs

The initial programme theory suggested that when CPs with pre-existing good communication skills and experience working in a mental health setting were provided with the intervention resources they would engage with, understand how to, and have the skills to work collaboratively with SUs. Contact data provided by CPs demonstrated a range of durations, frequencies, content, and media used when meeting with SUs. Additionally, 88.6% of SU survey responders reported that their CP really listened and understood them; 89.7% that they trusted their CP and felt positive working with them. Together these are indicative of CPs delivering an intervention that had been individualised to the SU and that the CPs were working collaboratively with the SUs.

However, session recording and interview data demonstrated that there were differences in the consistency with which Grace and other CPs were able to develop a collaborative relationship with SUs. Once Grace had developed the skills to create relationships, her session recordings, interview transcripts, and reflective practice logs demonstrated her doing so consistently in line with the model. Grace was able to do this regardless of needs of the SU. For example, in this session recording of CP Grace working with SU Keith she demonstrates a good understanding of what is important to Keith:

*that’s your ultimate goal, isn’t it, it’s to go back into business, but because things sort of fell apart the last time, you’re almost, you know, in a–is this going to sound really strange, but in a good position, really [laughs], to learn from your mistakes.* [CP Grace]

*Exactly*, *I said exactly the same to [CBT therapist] the other day*, *I can learn from what I did wrong last time* [SU Keith]

Analysis of session recordings and interviews show Nora developing collaborative relationships with some SUs. For example, SU Tina, who had challenging physical problems, a difficult spousal relationship, and was currently unemployed, reflects on a positive relationship with Nora:

*it was a chance to actually sound things out to somebody who a) wasn’t going to judge me, but b) could potentially direct me in whatever options that may be available. And working with one person, meant that I, once I’d developed that bond with them, it felt safe enough to accept that there was no hidden agenda* [SU Tina]

Unlike Grace, but like other CPs, the development of a collaborative relationship was not evident across all SUs with whom Nora worked. For example, Nora and Hannah both experienced a loss of engagement and withdrawal from SUs who perceived there was a loss of trust between the CP and the SU, and one SU (James) reported in interview that he found the intervention unhelpful because he did not feel listened to:

*It felt like what I was saying didn’t have much impact on the care partner or she felt maybe that wasn’t something she would or could go into further detail about* [SU James]

Analysis of the context of these SUs suggests that CPs sometimes had difficulty individualising the intervention and working in a patient-centred, recovery-focused manner, and instead delivered the intervention more mechanically. For example, analysis of SU context showed that Nora was able to work more collaboratively with SUs with greater socio-economic adversity. It was not clear whether these differences were related to the contextual factors of the CPs; it was not related to the expected contextual factors of pre-existing communication skills and experience of working in a mental health setting.

Cross-case analysis showed some indication that CPs were more able to work collaboratively with SUs who had similar needs to those they had worked with in previous roles or were of a similar socio-economic status to them. For example, Nora was more able to work collaboratively with those with very high levels of socioeconomic need (e.g., long-term unemployed, fragile housing and difficult personal relationships) as she had worked with similar needs before. She was less able to create a collaborative relationship with those who were for example, looking to move from paid employment to a more challenging role, or move from living with relatives to living independently. However, this was not considered increased fidelity when working with these higher need SUs, but rather an indication of mechanical-non-individualised care that overall had less fidelity to the model.

### 3.2 Coaching and goal setting

The initial programme theory suggested that when CPs were provided with the intervention resources they would engage with, understand how to, and have the skills to utilise a coaching and goal setting model with SUs. Again, pre-existing good communication skills and experience working in a mental health setting were expected to be facilitators in causing this outcome. 64.9% of SU survey respondents said that their CP helped them set goals to work on together. Although, the majority of SU survey respondents reported that their CP helped them to think about their physical (62.8%) or mental health (88.5%), less said that their CP helped them to act to improve their physical (42.5%) or mental health (73.7%). The SU contextual reasons for differences between thinking and acting are outside the scope of this paper, being part of the refinement the SU level part of the initial programme theory. Below explores reasons why delivery of the coaching and goal setting outcome varied.

In interview both SUs and CPs referred to goals and coaching within the context of the collaborative relationship and shared understanding. This suggests that fidelity to the delivery of coaching and goal setting was contingent on the ability to consistently create collaborative relationships and shared understandings with SUs. This allowed the CP to understand which goals might be of interest to the SU, what personal barriers there may be for the SU in achieving these, and how best to tackle any barriers:

*from meeting somebody to setting goals it’s about building up a rapport, listening to them and what matters to them, and then we look at what is it that they might like to change that would make things better for them. And that’s how, you know, they establish, really, what they would like to do.* [CP Nora]

For SUs, the coaching outcome was not perceived to be fully delivered if it did not match their individual needs. Matching these individual needs was contingent on having successfully created a collaborative relationship and shared understanding with the SU. SU James, who also felt his CP did not truly listen to him, reflects on the coaching he received not quite meeting his needs because he personally needed a more authoritarian approach:

*“Stick to your goal today, do it”, you know “Don’t forget”, or “I want to hear by the end of the day that you’ve done it”. Not that I want to, but it would be nice to hear that. It’s not a drill sergeant, obviously.[…] A bit more pressure, I guess.* [SU James]

This may account for some of the discrepancy between SUs thinking about goals and achieving goals. However, it should be noted that much of this discrepancy is due to SU contextual factors that are explored further in the refinement of the SU level programme theory.

### 3.3 Liaising with other services

In the initial programme theory when CPs are provided with the intervention resources they will engage with, understand how to, and have the skills to liaise with other services as appropriate according to the shared understanding and collaborative relationship between the SU and CP. Any liaison should be undertaken in a way that empowers SUs, rather than simply doing things for them and/or without consulting them. Where agreed with the SU this might involve sharing elements of the shared understanding with other practitioners. This is expected to be facilitated by CPs having previous experience of working in a mental health setting and having pre-existing good communication skills. Contact data provided by CPs demonstrated 143 incidents of signposting across 55 of the SUs; this is consistent with the SU survey data, where 47% of SUs reported receiving signposting to other services. CPs also reported discussing physical health, mental health, and social needs in the majority of meetings with SUs (92%, 92%, 95% respectively). Although these figures are indicative of discussion and signposting occurring, the qualitative case studies are more useful in drawing conclusions about whether the liaison element was being delivered as expected.

Qualitative data showed that, unlike some CPs, both Grace and Nora demonstrated good liaison with primary and secondary care practitioners, addressing issues such as mental health medication reviews and physical health problems; demonstrating good delivery in line with the model. Both CPs routinely made entries in primary and secondary care records, and undertook ad hoc liaison related to specific needs arising as a result of shared understandings with SUs. For example, interviews, reflective practice logs and session recordings demonstrated Grace liaising with primary and/or secondary care in relation to: COVID-19 shielding; COVID-19 positive diagnosis; Possible lithium poisoning; Arranging a smear test; Adjustment of pain medication; Changing the location of lithium blood tests to better suit the SU. However, across all CPs there were no instances of working with other providers to develop or elaborate or review the individuals as a whole or the ‘shared understanding’ they had developed. The extent to which liaison truly matched expected outcomes by being empowering for SUs and being based on the shared understanding/collaborative relationship, rather than a more traditional ‘fixing’ or ‘doing for’ mode, was not always clear. For example, Nora describes liaising with GPs about SUs, but it is not clear to what extent this is empowering the SU:

*there’s been one lady I was particularly concerned about, so I asked her permission to talk to the manager of the assisted living and she said yes. Oh, the manager was overwhelmed that I’d called her. We’ve involved the consultant and the GP, you know, just made them aware that things are not going well for this lady. I’ve got another lady that’s had to go through a change of medication from the consultant; conversations with the GP. I had another gentleman very irate ‘cos he should be on the vulnerable list and he hadn’t received his letter and he didn’t need to be on the vulnerable list, so I talked to the GP and she [the GP] was very happy to ring him and reassure him.* [CP Nora]

The initial programme theory stated that previous experience of working in a mental health setting would facilitate the liaison outcome. However, for some CPs previous work within a mental health setting was insufficient; these practitioners required an existing relationship with the person/place with which they were trying to liaise in order to feel successful, comfortable, and confident liaising with other bodies.

*I’ve had to continually keep repeating myself and almost trying to knock on the door and introduce myself [laughs], which is difficult.* [CP Sarah]

A ‘hunch’ as to whether this variation in fidelity was caused by previous training, role, or seniority was explored. Analysis across case studies showed that qualifications and previous job role did not impact the firing of these mechanisms, as they were not indicative of the expected contextual facilitator of previous skill and experience. For example, despite their different professional backgrounds, both Grace (band 6 mental health manager) and Nora (band 3 support worker) were confident and competent in establishing and maintaining relationships with primary and secondary care practitioners:

*Reception know exactly who I am, why I’m here and they’ve sorted out, um, a patient this morning, really quickly. Um, I’ve attended, with patients, to see the GP, I’ve attended, um, outpatient appointments with mental health services.* [CP Grace]

This suggests that delivery of the liaison element of the model is not affected by the professional background of the practitioner, but rather existing skills, experience, and relationships that are not qualification-based or hierarchical in nature.

### 3.4 Time to develop practice

The initial programme theory states that in response to the intervention resources two changes in reasoning are required for CPs to deliver the intervention: Understand and engage with the intervention; Understand how to and have the skills to deliver the intervention. Interviews, reflective logs, and session recordings showed that all CPs took time to create these required changes in reasoning. Therefore, fidelity to the initial programme theory improved over time.

*I learn with each session, to be quite honest with you, and what I tend to do is refresh things throughout the manual because, you know, delivering it is important, but at the same time adapting it to the people that you’re working with can be quite tricky.* [CP Nora]

Analysis of CP context demonstrated that the reasoning changes required happened in stages, with different CPs grasping different elements of the intervention at different time points. Which elements were understood first varied by CP, based on their previous job responsibilities and experience. For example, Nora, who had previously undertaken a lot of signposting work, had an early understanding of the intervention which focused on liaison across services. A deeper understanding of the collaborative relationship with the SUs, shared understanding and goal setting elements developed later in her practice. This has implications for interpreting the trial results: different CPs delivering different elements of the model with different levels of fidelity at different times. Additionally, SUs recruited towards the end of the trial or working with CPs who had a longer time in post are likely to have received an intervention with greater fidelity.

Nora’s initial understanding of the model:

*it had been recognised that there was a bit of a gap between the way primary care, secondary care [work], secondary care, primary care look after people from a sort of beginning to an ending or an ending to a beginning whichever way the transition goes. It just, you know, it needs perhaps more continuity and a smoother way of working.* [CP Nora]

Nora’s later understanding of the model:

*you look at it [SU progress] together [with the SU], you own it together, you spend time together devising the plan, you know, sort of motivating them towards identifying goals that they can work on within their day-to-day structure. But also it was about using the time that I’m with people, picking up on things that they’re–it’s concerning them and they’re wanting to do.* [CP Nora]

The initial programme theory stated that previous experience in working in a mental health setting would be a facilitator in understanding how and having the skills to deliver the PARTNERS model. However, the role of previous experience was more complicated than this. CPs reported that one of the reasons it took time to learn how to deliver the collaborative relationship and shared understanding elements of the model was that they had to ‘unlearn’ previous practice: Although CPs reported grasping the ethos of PARTNERS early on (i.e., understood and engaged with the intervention), it took longer to be able to put this understanding in to practice (i.e., understand how to and have the skills to deliver the intervention). For example, although CPs were excited and keen to work collaboratively with SUs using a coaching model, it was difficult in practice to move away from the familiar practices of working in a ‘one size fits all’ mechanistic way and a maternalistic/paternalistic ‘doing for’ approach; this took time.

This suggests previous experience working in a mental health setting is a contextual barrier to delivering these outcomes:

*After the first lot of training, um, it [sighs], oh dear, I mean, um, it felt–I felt quite–how shall I say–not scared, but quite worried that, you know, that you wouldn’t be delivering what was being asked for during the training. I suppose there could be all sorts of various reasons for that. One is that for quite some time beforehand, um, you didn’t have the opportunity to practise in that kind of way, because of the things that I’ve sort of mentioned, you know, implement[ing] targets and, um, paperwork, um, you know, all that type of stuff. And so, it almost felt as though we were starting all over again* [CP Grace]

A ‘hunch’ as to whether this unlearning was facilitated by previous training, role, or seniority was explored. Analysis of interviews regarding CP supervision and CP reflective practice logs show that this unlearning happened quicker where CPs engaged more in ongoing training and more fully utilised the opportunity to use reflective practice. This suggests that previous experience of continuous reflective practice, which may be less common in support worker roles, may be a facilitator in unlearning undesirable practice in order to create the collaborative relationship and shared understanding outcomes. This appeared to be due to increased familiarity with exploring and addressing any weaknesses in personal practice in non-support worker roles. This represents a development to the current programme theory with previous experience working in a mental health setting playing a more complex role than portrayed in the initial theory, instead functioning as both a barrier and a facilitator.

## 4. Discussion

This paper aimed to explore how, why, and under what circumstances intervention delivery matched the Realist initial programme theory for PARTNERS.

Delivery compared to model was overall good. However, as expected in a complex intervention [29], delivery varied. The Realist approach offered an opportunity to explore how, why and under what circumstances delivery varied [18]. The consistency of delivery of the collaborative relationship element of the model varied by practitioner [30], with some finding it more difficult to adapt to different SU contexts. Ability to deliver this element impacted on ability to deliver coaching to SUs. Delivery of the liaison element of the model was dependant on the practitioner contextual factors of pre-existing skills and relationships.

Quantitative and qualitative findings broadly agreed in this Realist evaluation of fidelity. Qualitative elements added helpful detail and depth as to how, why and under what circumstances delivery matched the initial programme theory. For example, highlighting the need for practitioners to have been in post for some time before having fully experienced the necessary changes in reasoning required to deliver the model as expected [26]. Fidelity methodologists have noted that this impact of practitioner experience was previously underexplored in fidelity science [29]. Qualitative data also highlighted system level challenges made it difficult to assess the impact of supervision and secondary care engagement mechanisms on practitioners delivering the intervention as expected; NHS pressures made it difficult to recruit, train, and retain supervisors for the practitioners.

These findings are helpful in interpreting the PARTNERS2 neutral trial findings [12]. Variation in delivery by practitioner and time in post, and difficulties implementing the supervision/secondary care engagement mechanism mean that caution should be applied when interpreting these trial findings. Those looking to implement similar models may wish to consider how to optimise practitioner training and other support to take account of the time needed to learn the model, and consider how to support practitioners in developing the relationship skills required to work collaboratively with SUs and successfully liaise across NHS boundaries. Any further implementation research regarding PARTNERS should investigate how to improve implementation of the supervision/secondary care engagement mechanism and how to optimise practitioner training to improve delivery of the model.

### 4.1 Strengths and limitations

The large volume of data, variety of data sources, and range of participants enabled a high degree of triangulation, increasing the rigour of this work. Meetings with stakeholders, including those with lived experience and their friends and family, contributed to the analysis process, establishing findings in the context of the NHS, lived and clinical experience, and wider literature.

Difficulties recruiting and retaining supervisors, and competing demands on supervisors’ time meant limited engagement by supervisors in training; often interim supervision had to be provided the researchers. Data provided by supervisors was also limited. Additionally, it should be noted that most CPs had volunteered/applied for this role with the support of the system in which they worked, which may have implications when interpreting the extent to which they engaged with the model to wider practitioner populations. This restricts the conclusions that can be drawn in relation to supervision and its impact on intervention fidelity.

## 5. Conclusion

Delivery of the PARTNERS intervention compared to model was generally good, but varied by practitioner and over time; some practitioners struggled to individualise the intervention and to liaise with other bodies. Trial results should be interpreted in light of this. Services implementing collaborative care should consider the time taken for practitioners to change practice and avoid expecting immediate change. Depending on individual inter-personal skills, some practitioners may require support and extra training to develop the ability to liaise across different providers and to create relationships with a wider range of people.

## Supporting information

S1 Checklist RASMESES reporting standards checklist. (DOCX)
